# Diagnosis of acute kidney injury and its association with in-hospital mortality in patients with infective exacerbations of bronchiectasis: cohort study from a UK nationwide database

**DOI:** 10.1186/s12890-016-0177-5

**Published:** 2016-01-19

**Authors:** Masao Iwagami, Kathryn Mansfield, Jennifer Quint, Dorothea Nitsch, Laurie Tomlinson

**Affiliations:** Department of Non-Communicable Disease Epidemiology, London School of Hygiene and Tropical Medicine, Keppel Street, London, WC1E 7HT UK; Department of Respiratory Epidemiology, Occupational Medicine and Public Health, National Heart and Lung Institute, Imperial College London, London, UK

**Keywords:** Acute kidney injury, Bronchiectasis, Clinical practice research datalink, Lower respiratory tract infection, Pneumonia

## Abstract

**Background:**

Many patients with bronchiectasis have recurrent hospitalisations for infective exacerbations. Acute kidney injury (AKI) is known to be associated with increased in-hospital mortality. This study examined the frequency of AKI, associated risk-factors, and the association of AKI with in-hospital mortality among patients with bronchiectasis.

**Methods:**

Anonymised data of patients with non-cystic fibrosis bronchiectasis from the UK Clinical Practice Research Datalink, linked to Hospital Episode Statistics, were used to identify hospitalisations with a primary diagnosis of lower respiratory tract infection (LRTI), from 2004 to 2013. After estimating the proportion of AKI diagnoses, a multivariable logistic regression model was constructed to investigate which background factors were associated with AKI. In-hospital mortality was compared between hospitalisations with and without an AKI diagnosis, with subsequent logistic regression analyses carried out for the association between AKI and in-hospital mortality.

**Results:**

Of 7804 hospitalisations due to LRTI observed in 3477 patients with bronchiectasis, 230 hospitalisations involved an AKI diagnosis, an average of 2.9 %. However, the percentage increased from less than 2 % in 2004 to nearly 5 % in 2013. After taking this temporal change into account, AKI was independently associated with older age, male sex, decreased baseline kidney function, previous history of AKI, and a diagnosis of sepsis. In-hospital mortality was 33.0 % (76/230) and 6.8 % (516/7574), in hospitalisations with and without AKI, respectively (*P* < 0.001). After adjustment for confounding factors, diagnosis of AKI remained associated with in-hospital mortality (Odds ratio 5.52, 95 % confidence interval: 3.62-8.42).

**Conclusions:**

Among people with bronchiectasis hospitalised for infective exacerbations, there is an important subgroup of patients who develop AKI. These patients have substantially increased in-hospital mortality and therefore greater awareness is needed.

## Background

Bronchiectasis, a syndrome of airway dilatation and bronchial wall thickening, has been increasing in prevalence in the community [1, 2]. A UK study showed that the period prevalence of non-cystic fibrosis (CF) bronchiectasis was 0.7 % over the 8-year period from 2004 to 2011, and that its overall incidence has increased from 18/100,000 in 2004 to 32/100,000 person-years at risk in 2011 [2]. Some patients with bronchiectasis experience infective exacerbations due to impaired respiratory drainage and immune function. Although short- and long-term use of antibiotics, such as nebulised gentamicin, has been reported to be effective in reducing airway inflammation [3, 4], hospital care is recommended for severe cases according to the British Thoracic Society guideline for non-CF bronchiectasis [5].

Acute kidney injury (AKI), previously known as acute renal failure, can be precipitated by a range of triggers and is common among hospitalised patients [6]. AKI is associated with a poorer prognosis, including among patients with community acquired pneumonia [6–8]. It is anticipated that patients with bronchiectasis would have a number of risk factors for AKI including the development of sepsis as a result of infectious exacerbations and frequent exposure to antibiotics such as gentamicin, a drug with nephrotoxic potential.

However, to the best of our knowledge, there has been no published study of AKI among patients with bronchiectasis. Therefore, using a UK nationwide outpatient database linked to inpatient data, we aimed to investigate the proportion of patients that develop AKI, the risk factors and the association between AKI and in-hospital mortality among patients with infective exacerbations of bronchiectasis.

## Methods

### Data source

The Clinical Practice Research Datalink (CPRD) is the National Health Service (NHS)’s observational data and interventional research service [9]. The database includes the following data: patient demographics; coded diagnoses with dates; prescriptions; laboratory test results; and referrals made by general practitioners (GPs). The CPRD can be linked with Hospital Episode Statistics (HES), which contains details of all admissions to NHS hospitals in England [10]. HES data consist of main and subsidiary diagnoses, coded using the 10th revision of International Classification of Disease (ICD-10) [11], and admission and discharge dates with discharge status (dead or alive). Approximately 60 % of CPRD practices have consented to linkage with HES. For this study, we used the CPRD linked to the latest HES dataset between 1st April 1997 and 31st March 2014. This dataset includes nearly 10 million patient records in 398 GP practices. Identification of diseases and prescriptions was based on diagnosis codes (Read codes) and product codes in the CPRD and ICD-10 codes in the HES. The list of Read codes and products codes used for this study is available on request from the authors.

Informed consent was waived for CPRD data because the data are anonymously extracted. Ethical approval for this study was obtained from the Independent Scientific Advisory Committee, which oversees research involving CPRD data (Protocol 14_127RA), as well as from the London School of Hygiene and Tropical Medicine ethics committee (reference: 7355).

### Study population and identification of hospital episodes

We identified adult patients with non-CF bronchiectasis within the CPRD at any time in the 10-year period between 1st April 2004 and 31st March 2014. The start date was chosen because the first unified criteria for AKI were established in 2004 [12]. Included patients began follow-up at the latest time-point of: the patient’s 18th birthday; the date bronchiectasis was first coded, one year after patients’ registration at a practice, the date the practice reached CPRD quality control standards; or 1st April 2004. The follow-up period ended at the first time-point of: death; the patient leaving the practice; the last data collection from the practice; or 31st March 2014.

We defined infective exacerbation of bronchiectasis as hospitalisation with lower respiratory tract infections (LRTIs). ICD-10 codes suggestive of LRTI (including pneumonia, but excluding chronic obstructive pulmonary disease (COPD) exacerbation) were pre-specified: J12, J13, J14, J15, J16, J17, J18, J20, J21, J22, and J47. We included all hospitalisations with these ICD-10 codes as a primary diagnosis, during the follow-up period for each patient. We excluded hospitalisations after the development of end-stage renal disease (ESRD), or chronic kidney disease (CKD) stage 5 (estimated glomerular filtration rate (eGFR) <15 mL/min/1.73 m^2^) [13].

### Variables

The gold-standard criteria for defining AKI are changes in creatinine in relation to baseline, or changes in urine output. The first global criteria for AKI (acute renal failure at that time) were established in 2004 [12]: We did not have access to biochemical data during hospitalisation and therefore defined hospitalisations with AKI using the following ICD-10 codes: N17.0, N17.1, N17.2, N17.8 and N17.9, regardless of the code position (i.e., secondary diagnosis or lower order, while a primary diagnosis had to be a LRTI code).

We pre-specified potential risk factors for a diagnosis of AKI which could be confounding factors in the association between AKI diagnosis and in-hospital mortality. In addition to sex and smoking status, we included 10 out of 14 general risk factors for AKI (Table 1), which were proposed in the recent National Institute for Health and Care Excellence (NICE) guidance [14]. For baseline kidney function, we calculated eGFR from serum creatinine value recorded in the CPRD, using Chronic Kidney Disease Epidemiology Collaboration equation [15], and categorized them according to NICE guidance for CKD [13]. Finally, we included co-diagnosis of COPD, because there is a close association between COPD and bronchiectasis [16, 17], and COPD is associated with AKI [18].

**Table 1:** List of risk factors for acute kidney injury selected and defined for the study

Risk factors proposed in NICE guidance for AKI	Definition in the current study
- Chronic kidney disease (eGFR < 60 mL/min/1.73 m^2^)	- Outpatient eGFR^a^ categorised into groups: > 60, 45–60, 30–45, and 15–30 mL/min/1.73 m^2^, and “No measurement” allocated if serum creatinine had not been measured for the past 2 years prior to the hospitalisation for LRTI.
- Heart failure	- Diagnosis of heart failure, recorded in CPRD before the hospitalisation for LRTI
- Liver disease	- Diagnosis of cirrhosis in CPRD, as a representative code for liver disease
- Diabetes	- Diagnosis of diabetes in CPRD
- History of AKI	- Diagnosis of AKI in CPRD or any hospitalisations with diagnosis of AKI in HES
- Oliguria (urine output < 0.5 mL/kg/h)	(Not obtained in the database)
- Neurological or cognitive impairment or disability, which may mean limited access to fluids because of reliance on a carer	- Diagnosis of dementia in CPRD, as a representative condition
- Hypovolaemia	(Not obtained in the database)
- Use of drugs with nephrotoxic potential (such as NSAIDs, aminoglycosides, ACEI, ARBs, and diuretics)	- NSAIDs, aminoglycosides (products for injection and nebuliser), ACEI or ARBs, and diuretics (loop, thiazide, and potassium-sparing diuretics), prescribed for the past 3 months prior to the hospitalisation for LRTI
- Use of iodinated contrast agents	(Not obtained in the database)
- Symptoms or history of urological obstruction or conditions that may lead to obstruction	- Diagnosis of prostatic hypertrophy in CPRD, as a representative condition
- Sepsis	- Sepsis identified as an additional diagnosis code of sepsis in HES during the hospitalisation for LRTI
- Deteriorating early warning scores	(Not obtained in the database)
- Age (≥ 65 years)	- Age categorised into groups: < 65, 65–74, 75–84, and > 85

ACEI, angiotensin converting enzyme inhibitor; AKI, acute kidney injury; ARB, angiotensin II receptor blocker; CPRD, Clinical Practice Research Datalink; eGFR, estimated glomerular filtration rate; LRTI, lower respiratory tract infection; HES, Hospital Episode Statistics; NICE, National Institute for Health and Care Excellence; NSAIDs, non-steroidal anti-inflammatory drugs

^a^Based on the most recent creatinine measurement, excluding those just before admission (within 28 days)

### Statistical analysis

We calculated the proportion of patients diagnosed with AKI among hospitalisations with LRTI overall, and then by financial year, considering that the identification of AKI in hospitals and the recording practice in the HES may have changed over time. For characteristics of hospitalisations with and without AKI, descriptive statistics were presented as percentages for all variables, with Chi-square (*χ*2) tests to examine differences between groups. Multivariable logistic regression analysis was then performed to examine which factors were associated with the diagnosis of AKI, taking year into account. In-hospital mortality was then compared between hospitalisations with and without AKI, with *χ*2 tests. Kaplan-Meier survival curves were plotted. Finally, we conducted multivariable logistic regression analyses of the association between AKI diagnosis and in-hospital mortality. Following an estimation of age-sex adjusted odds ratio, all the covariates defined above were entered into the multivariable regression model. In order to take into account the fact that many patients were repeatedly hospitalised, a random effect model was used for all logistic regression analyses. A small number of cases with missing smoking status were excluded. A *P* value of < .05 was inferred as statistically significant. All statistical analyses were conducted using Stata 13 software (Stata Corp, Texas).

### Sensitivity analysis

To treat potential uncertainties of the data in CPRD and HES, we conducted sensitivity analyses under the following three scenarios to examine whether there would be a marked change in the conclusion regarding the association between AKI diagnosis and in-hospital mortality: (i) we excluded patients without baseline eGFR data, because absence of information on outpatient kidney function may be associated with whether or not AKI is diagnosed by the responsible clinicians; (ii) we excluded patients with co-diagnosis of COPD, because bronchiectasis diagnosed in the context of COPD follow-up may be different from bronchiectasis diagnosed on its own; (iii) we limited the hospitalisations to those with a primary diagnosis of pneumonia (ICD-10 codes J12, J13, J14, J15, J16, J17, and J18), because these cases are likely to be more severe and definitive among the cases of LRTI we have included.

## Results

**Fig. 1:**
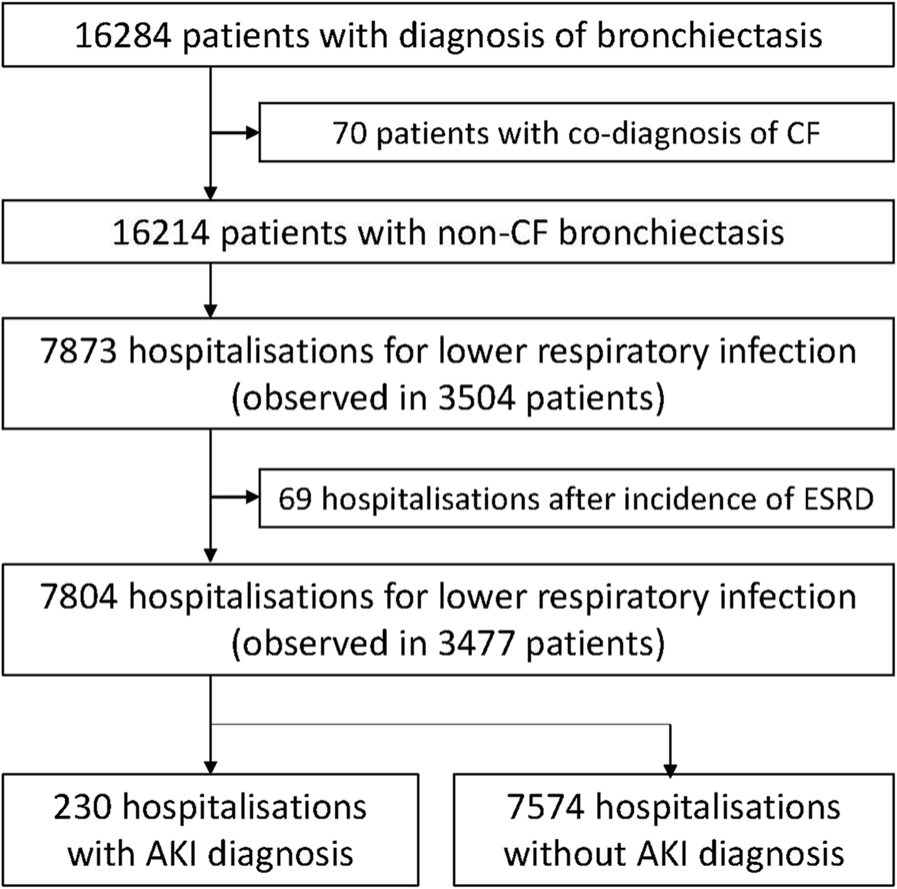
Flow chart for the selection of eligible hospitalisations with and without acute kidney injury diagnosis. AKI, acute kidney injury; CF, cystic fibrosis; ESRD, end stage renal disease

**Fig. 2:**
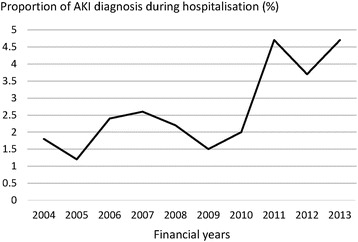
Annual change in the proportion of acute kidney injury diagnosis during hospitalisation. AKI, acute kidney injury

Figure 1 shows the workflow by which eligible hospital episodes were identified. Of 16,214 patients with non-CF bronchiectasis registered in the HES-linked CPRD between 01/04/2004 and 31/3/2014, 3504 patients had at least one hospitalisation for LRTI, a total of 7873 hospitalisations. The median number of hospitalisations per patient during the follow-up period was two, with an interquartile range of 1–4. We excluded 69 hospitalisations that occurred after the development of ESRD. Of the remaining 7804 hospital episodes, 230 hospitalisations involved AKI diagnoses, accounting for 2.9 % (95 % confidence interval [CI]: 2.6-3.3 %). Fig. 2 illustrates the annual change in the proportion of AKI diagnosis from 1st April 2004 to 31st March 2014. The percentage increased, from 1.8 % (8/475 hospitalisations) in 2004 to 4.7 % (54/1096 hospitalisations) in 2013.

Table 2 compares patient characteristics between hospitalisations with and without AKI. Patients with an AKI diagnosis were more likely to be male and were older than those without an AKI diagnosis. The proportion of patients with a history of current or previous smoking and co-diagnosis of COPD were higher in those with an AKI diagnosis. The baseline kidney function was worse in patients admitted with AKI: nearly half had an eGFR less than 60 mL/min/1.73 m^2^ in the group with an AKI diagnosis, while only around 15 % of patients without AKI had an eGFR less than 60 mL/min/1.73 m^2^. Previous history of: heart failure, diabetes, AKI, dementia and prostatic hypertrophy, as well as recent prescription of: non-steroidal anti-inflammatory drugs (NSAIDs), aminoglycoside nebuliser, angiotensin converting enzyme inhibitors (ACEI) or angiotensin II receptor blockers (ARBs), diuretics, and a concurrent diagnosis of sepsis, were more frequent in those with AKI than in those without AKI. Table 3 shows the results of multivariable logistic regression analysis. Older age, male sex, decreased baseline eGFR, history of AKI, and sepsis diagnosis, were independently associated with the diagnosis of AKI.

**Table 2:** Comparison of patient characteristics between hospitalisations for lower respiratory tract infection with and without acute kidney injury

	Hospitalisations with AKI diagnosis (*N* = 230) (%)	Hospitalisations without AKI diagnosis (*N* = 7574) (%)	*P* Value
Age (years)			< 0.001
< 65	10.9	31.4	
65-74	22.2	29.2	
75-84	41.7	27.9	
≥ 85	25.2	11.5	
Sex (Male)	53.0	36.3	< 0.001
Smoking history			0.037
Non-smoker	28.7	37.7	
Ex-smoker	59.6	53.4	
Current smoker	11.3	8.6	
Missing	0.4	0.3	
Co-diagnosis of COPD	60.4	50.0	0.002
Outpatient eGFR (mL/min/1.73 m^2^)			<0.001
> 60	33.0	53.8	
45-60	17.4	11.0	
30-45	21.3	4.2	
15-30	10.0	1.3	
No measurement	18.3	29.8	
History of AKI	20.4	4.4	< 0.001
Chronic conditions			
Heart failure	19.6	9.0	< 0.001
Cirrhosis	0.9	0.6	0.638
Diabetes	26.1	13.1	< 0.001
Dementia	4.8	2.9	0.107
Prostatic hypertrophy	17.0	7.5	< 0.001
Drugs with nephrotoxic potential			
NSAIDs	28.3	22.5	0.038
Aminoglycoside nebuliser	1.3	0.1	0.001
Aminoglycoside injection	1.3	0.7	0.227
ACEI or ARBs	35.2	21.9	< 0.001
Diuretics	44.8	29.5	< 0.001
Sepsis diagnosis	12.2	1.0	< 0.001

ACEI, angiotensin converting enzyme inhibitor; AKI, acute kidney injury; ARB, angiotensin II receptor blocker; COPD, chronic obstructive pulmonary disease; eGFR, estimated glomerular filtration rate; NSAIDs, non-steroidal anti-inflammatory drugs

**Table 3:** Multivariable logistic regression analysis for factors associated with diagnosis of acute kidney injury

	Adjusted odds ratio^a^	95 % confidence interval
Age (years old)		
<65	Reference	
65-74	1.49	0.87 – 2.57
75-84	2.26	1.34 – 3.80
≥85	2.64	1.48 – 4.68
Sex (Male/Female)	1.75	1.23 – 2.49
Smoking history		
Non-smoker	Reference	
Ex-smoker	0.94	0.65 – 1.35
Current smoker	1.50	0.87 –2.58
Co-diagnosis of COPD (Yes/No)	1.18	0.85 – 1.63
Outpatient eGFR (mL/min/1.73 m^2^)		
> 60	Reference	
45-60	2.44	1.54 – 3.86
30-45	7.42	4.46 – 12.34
15-30	10.73	5.35 – 21.53
No measurement	1.55	0.99 – 2.42
History of AKI (Yes/No)	1.93	1.23 – 3.03
Chronic conditions (Yes/No)		
Heart failure	1.06	0.69 – 1.63
Cirrhosis	1.50	0.25 – 9.16
Diabetes	1.38	0.94 – 2.02
Dementia	0.92	0.44 – 1.93
Prostatic hypertrophy	1.28	0.79 – 2.08
Drugs with nephrotoxic potential (Yes/No)		
NSAIDs	0.95	0.66 – 1.36
Aminoglycosides nebuliser	6.15	0.43 – 87.59
Aminoglycosides injection	3.90	0.98 – 15.58
ACEI or ARBs	1.34	0.94 – 1.92
Diuretics	1.19	0.84 – 1.70
Sepsis diagnosis (Yes/No)	18.32	10.04 – 33.41

ACEI, angiotensin converting enzyme inhibitor; AKI, acute kidney injury; ARB, angiotensin II receptor blocker; COPD, chronic obstructive pulmonary disease; eGFR, estimated glomerular filtration rate; NSAIDs, non-steroidal anti-inflammatory drugs

^a^Additionally adjusted by financial year of hospitalisation

**Fig. 3:**
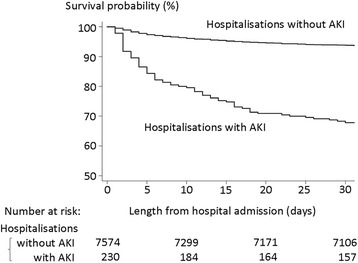
Kaplan-Meier survival curves by the status of acute kidney injury diagnosis. AKI, acute kidney injury

Overall in-hospital mortality was 7.6 % (592/7804 hospitalisations). In hospitalisations with and without an AKI diagnosis, in-hospital mortalities were 33.0 % (76/230) and 6.8 % (516/7574), respectively (*P* < 0.001). Kaplan-Meier survival curve by AKI status demonstrates that patients with an AKI diagnosis had higher cumulative mortality, compared with those without an AKI diagnosis (Fig. 3). Multivariable logistic regression revealed that AKI diagnosis was positively associated with in-hospital mortality, with an age-sex adjusted odds ratio of 6.72 (95 % CI: 4.45-10.15), and an adjusted odds ratio of 5.52 (95 % CI: 3.62-8.42) after adjustment for all the covariates (Table 4). Table 4 also shows the results of sensitivity analyses. A positive association between AKI diagnosis and in-hospital mortality was consistently demonstrated in the three different scenarios.

**Table 4:** Association between diagnosis of acute kidney injury and in-hospital mortality: main analysis and sensitivity analyses

	In-hospital mortality		Odds ratio (95 % CI) of AKI diagnosis for in-hospital mortality	
	Hospitalisations with AKI	Hospitalisations without AKI	adjusted by age and sex	adjusted by all the confounding factors^a^
Main analysis (*N* = 7804)	33.0 % (76/230)	6.8 % (516/7,574)	6.72 (4.45-10.15)	5.52 (3.62-8.42)
Sensitivity analysis				
(i) Excluding patients with no outpatient creatinine measurement (*N* = 5506)	31.9 % (60/188)	7.9 % (420/5318)	5.83 (3.71 – 9.15)	4.61 (2.90 – 7.33)
(ii) Excluding patients with co-diagnosis of COPD (*N* = 3882)	29.7 % (27/91)	6.4 % (244/3791)	5.14 (2.59 – 10.21)	4.24 (2.12 – 8.47)
(iii) Hospitalisations limited to pneumonia diagnosis among LRTI (*N* = 2400)	43.1 % (66/153)	17.5 % (392/2247)	4.13 (2.63 – 6.46)	3.72 (2.34 – 5.89)

AKI, acute kidney injury; COPD, chronic obstructive pulmonary disease; CI, confidence interval; LRTI, lower respiratory tract infection

^a^All the factors shown in Table 2, in addition to financial year of hospitalisation

## Discussion

This study examined the burden of AKI among patients with non-CF bronchiectasis. Among approximately 16,000 patients with a diagnosis of non-CF bronchiectasis, over 20 % experienced at least one hospitalisation for LRTI during the observation period, much higher than that observed in the general population [19]. Of those who developed AKI during hospitalisation for LRTI, one third died. The strong association of AKI diagnosis with mortality (fully adjusted odds ratio 5.52 (95 % CI: 3.62-8.42)) has important implications for the clinical care of patients with bronchiectasis.

Given the high prevalence of risk factors for AKI among patients with bronchiectasis, the incidence of AKI found in this study was surprisingly low. A previous study, which defined AKI based on changes in serum creatinine values [12], reported that the incidence of AKI determined at admission was 18 % among patients hospitalised for community-acquired pneumonia [8]. Another study in the US found that 34 % of patients with community acquired pneumonia developed AKI during hospitalisation [7]. The diagnosis of AKI in this study depended upon recognition and diagnosis by the responsible clinicians, as well as subsequent coding of that diagnosis. A lower incidence of AKI obtained by coded diagnosis compared to that based on biochemical values is well established [20], but the size of the discrepancy in this study suggests that awareness of, and therefore diagnosis of, AKI may be still low among clinicians caring for this patient group. The increased incidence of AKI over time is likely to reflect at least in part growing awareness of the condition among clinicians, influenced by the publication of international guidelines and awareness campaigns [12, 21, 22].

Previously identified risk factors including older age, male sex, baseline kidney function, previous history of AKI, and sepsis diagnosis were independently associated with AKI diagnosis in this setting. The strongest association was for patients concurrently diagnosed with sepsis, with an odds ratio of 18.32 (95 % CI: 10.04-33.41), compatible with findings in previous studies [7, 8], and pathophysiological considerations [23]. Similarly CKD, known to be a major risk factor for AKI [24–26], was associated with increased odds of AKI in a stepwise fashion according to more advanced CKD stages.

To our knowledge this is the first study examining the diagnosis and outcome of AKI among patients with bronchiectasis, and has a number of strengths including a large, nationally representative sample of patients over a ten year period. The linkage between the outpatient and inpatient data allowed for quantification of the effect size of a number of risk factors and adjustment for important confounders of the association between AKI and in-hospital mortality.

Several limitations of this study must be acknowledged. Firstly, it uses routinely collected data and some variables may be misclassified. The diagnosis of bronchiectasis is predominantly made by respiratory physicians based on objective findings such as a chest CT scan, and subsequently entered into the primary care record by a GP [5, 27]. Therefore, the specificity of bronchiectasis diagnosis is expected to be high. However, no validation study between CPRD and patient records for bronchiectasis has been conducted to date. The definition of AKI is based on the presence of a relevant ICD-10 code in HES, and was not validated by creatinine values or urine volume. However, a single centre study in the UK demonstrated that, where present, an AKI code in HES had a 95 % positive predictive value compared with current biochemical definitions of AKI [28]. In Scotland, the proportion of biochemical cases of AKI also identified by coding increased markedly with greater severity of AKI [20]. Therefore while this study may have detected a minority of true AKI cases, those identified are likely to be valid and at the severe end of the spectrum. Similarly, it is likely that patients identified to have sepsis from HES coding data were more severe cases. Secondly, although we were able to adjust for many known risk factors for AKI, unmeasured confounding is possible. For example, access to local nephrology services could have affected both the likelihood of being diagnosed with AKI as well as in-hospital mortality [29]. Finally, although this study has the largest sample size examined to date, the statistical power was low for identification of risk factors with small effect sizes, and this may partly explain why variables such as the prescription of drugs with nephrotoxic potential were not identified as independent risk factors for AKI.

## Conclusions

This nationwide study is the first to investigate the risk factors for and outcomes of AKI among patients with bronchiectasis hospitalised for infective exacerbations. AKI was infrequently diagnosed but was strongly associated with mortality. A national programme is currently underway to improve outcomes from this preventable and treatable condition [30]. These results suggest that there needs to be a greater awareness of, and possibly better treatment of AKI among clinicians caring for patients with bronchiectasis to improve these poor outcomes.

## Abbreviations

ACEI: angiotensin converting enzyme inhibitor
AKI: acute kidney injury
ARB: angiotensin II receptor blocker
CF: cystic fibrosis
CKD: chronic kidney disease
COPD: chronic obstructive pulmonary disease
CPRD: Clinical Practice Research Datalink
eGFR: estimated glomerular filtration rate
ESRD: end-stage renal disease
GP: general practitioner
HES: Hospital Episode Statistics
ICD-10: 10th revision of International Classification of Disease
LRTI: lower respiratory tract infection
NHS: National Health Service
NICE: National Institute for Health and Care Excellence
NSAIDs: non-steroidal anti-inflammatory drugs
*χ*2: chi-square
